# Prediction of clinical risk assessment and survival in chronic obstructive pulmonary disease with pulmonary hypertension

**DOI:** 10.1002/ctm2.1702

**Published:** 2024-06-11

**Authors:** Dansha Zhou, Chunli Liu, Lan Wang, JiFeng Li, Yating Zhao, Zheng Deng, Chi Hou, Yingyun Fu, Qian Jiang, Ning Lai, Rui Zhang, Weici Feng, Chuhui Gao, Xiang Li, Mei Jiang, Xin Fu, Jiyuan Chen, Wei Hong, Lei Xu, Wenjun He, Jinming Liu, YuanHua Yang, Wenju Lu, Nanshan Zhong, Yunshan Cao, Jian Wang, Yuqin Chen

**Affiliations:** ^1^ State Key Laboratory of Respiratory Diseases, National Center for Respiratory Medicine, Guangdong Key Laboratory of Vascular Diseases, National Clinical Research Center for Respiratory Diseases, Guangzhou Institute of Respiratory Health the First Affiliated Hospital of Guangzhou Medical University Guangzhou Guangdong China; ^2^ Department of Pulmonary Circulation, Shanghai Pulmonary Hospital Tongji University School of Medicine Shanghai China; ^3^ Department of Respiratory and Critical Care Medicine, Beijing Chao‐Yang Hospital Capital Medical University Beijing China; ^4^ Department of Cardiology Gansu Provincial Hospital Lanzhou Gansu China; ^5^ The First People's Hospital of Yunnan Kunming Yunnan China; ^6^ Department of Neurology Guangzhou Women and Children's Medical Center Guangzhou Guangdong China; ^7^ Department of Pulmonary and Critical Care Medicine, Shenzhen Institute of Respiratory Disease Shenzhen Institute of Respiratory Disease,Shenzhen People's Hospital ( The Second Clinical Medical College，Jinan University；The First Affiliated Hospital, Southern University of Science and Technology） Shenzhen Guangdong China; ^8^ GMU‐GIBH Joint School of Life Sciences Guangzhou Medical University Guangzhou Guangdong China; ^9^ Department of Pulmonary and Critical Care Medicine The Affiliated Hospital of Inner Mongolia Medical University, Inner Mongolia Autonomous Region Hohhot China; ^10^ Heart, Lung and Vessels Center, Sichuan Provincial People's Hospital University of Electronic Science and Technology of China Chengdu Sichuan China; ^11^ Guangzhou Laboratory Guangzhou International Bio Island Guangzhou Guangdong China; ^12^ Section of Physiology, Division of Pulmonary, Critical Care and Sleep Medicine University of California, San Diego La Jolla California USA

**Keywords:** COPD, nomogram, pulmonary hypertension, survival

## Abstract

**Background:**

Patients with pulmonary hypertension (PH) and chronic obstructive pulmonary disease (COPD) have an increased risk of disease exacerbation and decreased survival. We aimed to develop and validate a non‐invasive nomogram for predicting COPD associated with severe PH and a prognostic nomogram for patients with COPD and concurrent PH (COPD–PH).

**Methods:**

This study included 535 patients with COPD–PH from six hospitals. A multivariate logistic regression analysis was used to analyse the risk factors for severe PH in patients with COPD and a multivariate Cox regression was used for the prognostic factors of COPD–PH. Performance was assessed using calibration, the area under the receiver operating characteristic curve and decision analysis curves. Kaplan–Meier curves were used for a survival analysis. The nomograms were developed as online network software.

**Results:**

Tricuspid regurgitation velocity, right ventricular diameter, N‐terminal pro‐brain natriuretic peptide (NT‐proBNP), the red blood cell count, New York Heart Association functional class and sex were non‐invasive independent variables of severe PH in patients with COPD. These variables were used to construct a risk assessment nomogram with good discrimination. NT‐proBNP, mean pulmonary arterial pressure, partial pressure of arterial oxygen, the platelet count and albumin were independent prognostic factors for COPD–PH and were used to create a predictive nomogram of overall survival rates.

**Conclusions:**

The proposed nomograms based on a large sample size of patients with COPD–PH could be used as non‐invasive clinical tools to enhance the risk assessment of severe PH in patients with COPD and for the prognosis of COPD–PH. Additionally, the online network has the potential to provide artificial intelligence‐assisted diagnosis and treatment.

**Highlights:**

- A multicentre study with a large sample of chronic obstructive pulmonary disease (COPD) patients diagnosed with PH through right heart catheterisation.
- A non‐invasive online clinical tool for assessing severe pulmonary hypertension (PH) in COPD.
- The first risk assessment tool was established for Chinese patients with COPD–PH.

## Graphical abstract

Overview of patient screening, data collection, non‐invasive risk assessment and prognostic nomogram development and validation for COPD–PH.

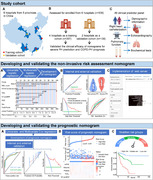

## 1 INTRODUCTION

Chronic obstructive pulmonary disease (COPD) is an increasing global health burden characterised by progressive irreversible airway restriction.^1^ COPD affects approximately 400 million people and is the third leading cause of death worldwide.^2^, ^3^ Pulmonary hypertension (PH) is a complication of COPD with an essential role in its progression to cardiopulmonary disease. Patients with COPD and concurrent PH (COPD–PH) have an increased risk of disease exacerbation and decreased survival.^4^ Many studies have reported a 30%–70% prevalence of PH in patients with COPD.^5^, ^6^

Notably, the lungs in patients with COPD and severe PH typically appear to have specific histological patterns, which differ from those observed in patients with COPD and moderate PH or without PH.^7^ The latest guidelines recommend individualised treatment for patients with suspected severe PH.^8^ Right heart catheterisation (RHC) is the gold standard for diagnosing PH, but it is invasive and risky.^9^, ^10^ Few studies have focused on non‐invasive examinations and clinical features for the stratification of patients with COPD–PH. Additionally, developing disease management tools that are easy to operate, scalable and have predictive value for chronic diseases such as COPD–PH, which require long‐term disease management in primary care units, is difficult. Therefore, developing non‐invasive tools for early diagnosis, monitoring and follow‐up of patients with COPD–PH is important and has clinical value. These tools could promote more precise prevention and personalised health management of COPD–PH and maximise cost‐effectiveness.

## 2 METHODS

### 2.1 Study population

In this retrospective, multicentre study of risk assessment and prognostic prediction, we collected the clinical data of 3012 patients with COPD and a tricuspid regurgitation velocity (TRV) >2.8 m/s on echocardiography. The data were collected from six hospitals in China from December 2008 to July 2021. According to the 2022 European Society of Cardiology/European Respiratory Society Guidelines for the diagnosis and treatment of PH,^11^ severe PH was defined as mean pulmonary arterial pressure (mPAP) >20 mm Hg with pulmonary vascular resistance (PVR) >5 WU.

### 2.2 Inclusion and exclusion criteria

The inclusion criteria for the study were as follows: (i) age >18 years; (ii) a diagnosis of COPD based on the predictive value of forced expiratory volume in 1 s (FEV1)/forced vital capacity (FVC) ratio <70% and FEV1≤80% determined by pulmonary function tests^12^; (iii) peak TRV (important variable for assigning the echocardiographic probability of PH) >2.8 m/s^11^; (iv) at least one pulmonary function test and echocardiography completed in the same hospital within 6 months; and (v) availability of RHC data at the diagnosis of PH showing an mPAP >20 mm Hg.^11^ The main exclusion criteria were as follows: (i) incomplete echocardiographic data; (ii) the presence of congenital heart disease and left heart disease; (iii) a history of pulmonary diseases, such as pulmonary embolism, interstitial pulmonary disease, active pulmonary tuberculosis and severe bronchiectasis; and (iv) a history of renal insufficiency, haematological diseases, rheumatic immune diseases, immunodeficiencies, or hyperthyroidism. The study was conducted in accordance with the Declaration of Helsinki and Good Clinical Practice guidelines. The requirement for informed consent was waived because of the retrospective nature of the study.

### 2.3 Cohort definition

Among the 3012 patients, 535 passed quality control and met the study's eligibility criteria. Of the 535 patients, 397 were from four hospitals in China, and they served as a training cohort to construct a non‐invasive nomogram for predicting the risk of severe PH in COPD. Additionally, 138 patients were from two additional hospitals and they served as an external validation cohort. Of the 535 patients, 76 (14.2%) were lost to follow‐up. The remaining 334 patients from the training cohort comprised the follow‐up cohort for constructing the nomogram to predict overall survival (OS). The remaining 125 patients from the validation cohort were used to assess the prognostic nomogram.

### 2.4 Statistical analysis

Univariate and multivariate logistic regression analyses were performed to identify the independent risk factors for severe PH in COPD. Univariate Cox models were used to assess the association between each variable and survival. Multivariate Cox regression models were used to analyse variables selected by clinical relevance. The performance of the nomogram was evaluated using the area under the curve (AUC) of the receiver operating characteristic (ROC) curve, calibration plots and decision curve analysis (DCA). The discrimination ability was evaluated by AUC of the ROC curve.^13^ The AUC value of >0.7 suggested good discrimination ability of the nomogram.^14^ Calibration plots were used to evaluate the calibration ability between the predicted and actual probability for each patient included in the nomogram model using the Hosmer–Lemeshow test. The *p* value of the Hosmer–Lemeshow test was >0.05, which indicated that the model had a high goodness of fit.^15^ The 45° straight line represents the perfect match between the actual and nomogram‐predicted probabilities.^15^ The DCA was used to evaluate the clinical benefits and utility of the nomogram.^16^ The distribution and difference in the logistics regression nomogram between severe PH and non‐severe PH were analysed and compared by violin plot analysis. The OS endpoint was calculated from the diagnosis to death or the last follow‐up date in July 2022. Data from patients alive at the last follow‐up were censored. Web servers were built based on validated nomograms to facilitate their use. The sample size estimation of the prediction model in this study was performed as previously described.^17^ In all analyses, *p* < 0.05 was considered statistically significant.

The data collection and detailed statistical analyses are described in the Supporting Information Appendix. The patient selection process and outcomes are shown in Supporting Information Figure E1.

## 3 RESULTS

### 3.1 Patient and disease characteristics

The total population included patients with COPD and non‐severe (*n* = 254) or severe PH (*n* = 281). Females with severe PH and COPD were younger than males with severe PH and COPD (59 years, interquartile range [IQR]: 48–66 vs. 64 years, IQR: 57–71, *p* < 0.001) and had better survival (*p *= 0.012). In the whole population, training cohort and validation cohort, the median ages of patients with COPD–PH were 64 (IQR: 57–71), 64 (IQR: 57–71) and 64 (IQR: 57–70) years, respectively. The median follow‐up time was 28 (IQR: 11–49), 31 (IQR: 12–51) and 23 (IQR: 9–45) months in the whole population, follow‐up cohort and validation cohort, respectively. In the whole population, follow‐up cohort and validation cohort, the 3‐year OS of severe PH in COPD was 66.2% (95% confidence interval [CI], 59.5%–73.6%), 64.9% (95% CI, 57.1%–73.7%) and 70.3% (95% CI, 58.2%–84.9%), respectively. The demographic and clinical characteristics of the training and follow‐up cohorts are shown in Table 1 and Supporting Information Table E1, respectively.

**TABLE 1:** Demographic and clinical characteristics of patients with COPD–PH in training and validation cohorts.

Variable	Whole population (*n* = 535)	Training cohort (*n* = 397)	Validation cohort (*n* = 138)	*p* value
Sex, no. (%)				0.765
Female	180 (33.6)	135 (34.0)	45 (32.6)	
Male	355 (66.4)	262 (66.0)	93 (67.4)	
Smoking status, no. (%)				0.332
Non‐smoker	304 (56.8)	223 (56.2)	81 (58.7)	
Ex‐smoker	68 (12.7)	47 (11.8)	21 (15.2)	
Smoker	163 (30.5)	127 (32.0)	36 (26.1)	
Severity of PH, no. (%)				0.137
Non‐severe PH	254 (47.5)	196 (49.4)	58 (42.0)	
Severe PH	281 (52.5)	201 (50.6)	80 (58.0)	
NYHA functional class, no. (%)				0.061
I/II	232 (43.4)	184 (46.3)	48 (34.8)	
III	252 (47.1)	177 (44.6)	75 (54.3)	
IV	51 (9.5)	36 (9.1)	15 (10.9)	
GOLD stage, no. (%)				0.701
Stage I	30 (5.6)	24 (6.0)	6 (4.3)	
Stage II	122 (22.8)	92 (23.2)	30 (21.7)	
Stage III	212 (39.6)	159 (40.1)	53 (38.4)	
Stage IV	171 (32.0)	122 (30.7)	49 (35.5)	
Age, median (IQR), year	64.0 (57.0–71.0)	64.0 (57.0–71.0)	64.0 (57.0–70.0)	0.714
Smoking exposure, median (IQR), year	0.0 (0.0–30.0)	0.0 (0.0–30.0)	0.0 (0.0–30.0)	0.637
NT‐proBNP, median (IQR), ng/L	746.0 (216.0–1933.0)	741.0 (222.0–1851.0)	778.0 (210.0–2469.0)	0.516
Six‐minute walk test, median (IQR), m	336.0 (200.0–430.0)	343.0 (200.0–441.0)	327.0 (172.0–396.0)	0.086
FEV1, median (IQR), %	37.0 (26.0–53.0)	37.0 (27.0–53.0)	37.0 (25.0–51.0)	0.237
FEV1/FVC, median (IQR), %	55.0 (45.0–63.0)	56.0 (46.0–63.0)	53.0 (43.0–63.0)	0.202
Body mass index, median (IQR), kg/m^2^	21.4 (18.4–24.2)	21.4 (18.3–24.3)	21.6 (18.5–23.8)	0.556
Red blood cell count, median (IQR), ×10^12^/L	4.9 (4.2–5.5)	4.9 (4.1–5.5)	5.0 (4.4–5.6)	0.267
White blood cell count, median (IQR), ×10^9^/L	6.5 (5.0–8.4)	6.5 (5.1–8.5)	6.5 (4.8–8.3)	0.216
Haemoglobin, median (IQR), g/L	142.0 (126.0–162.0)	141.0 (124.0–161.0)	144.0 (129.0–171.0)	0.098
Platelet, median (IQR), ×10^9^/L	183.0 (143.0–226.0)	184.0 (145.0–231.0)	174.0 (141.0–212.0)	0.064
Aspartate transaminase, median (IQR), U/L	22.0 (18.0–29.0)	22.0 (17.0–30.0)	23.0 (19.0–29.0)	0.351
Alanine aminotransferase, median (IQR), U/L	20.0 (14.0–30.0)	20.0 (14.0–29.0)	22.0 (14.0–31.0)	0.199
Total bilirubin, median (IQR), µmol/L	15.0 (10.9–22.2)	15.0 (10.6–22.0)	15.2 (11.1–23.1)	0.650
Direct bilirubin, median (IQR), µmol/L	5.3 (3.3–9.1)	5.3 (3.3–9.0)	5.5 (3.4–9.5)	0.599
Total protein, median (IQR), g/L	65.0 (60.0–71.0)	66.0 (60.0–72.0)	64.0 (60.0–69.0)	0.122
Albumin, median (IQR), g/L	37.0 (34.0–40.0)	37.0 (34.0–40.0)	37.0 (34.0–40.0)	0.268
Creatinine, median (IQR), µmol/L	70.0 (55.0–87.0)	70.0 (55.0–88.0)	66.0 (55.0–85.0)	0.377
Blood urea nitrogen, median (IQR), mmol/L	6.3 (4.8–8.6)	6.4 (4.7–8.8)	6.0 (5.0–8.0)	0.351
D‐dimer, median (IQR), mg/L	647.0 (320.0–1093.0)	597.0 (320.0–1090.0)	671.0 (326.0–1103.0)	0.551
Total cholesterol, median (IQR), mmol/L	4.0 (3.4–5.0)	4.0 (3.5–5.0)	4.0 (3.3–5.0)	0.074
Triglyceride, median (IQR), mmol/L	1.0 (0.8–1.8)	1.0 (0.8–1.8)	1.0 (0.8–1.5)	0.353
HDL‐C, median (IQR), mmol/L	1.1 (1.0–1.5)	1.1 (1.0–1.5)	1.1 (1.0–1.4)	0.760
LDL‐C, median (IQR), mmol/L	2.5 (2.0–3.0)	2.5 (2.0–3.0)	2.5 (2.0–3.0)	0.707
Erythrocyte sedimentation rate, median (IQR), mm/h	29.0 (10.0–47.0)	30.0 (10.0–49.0)	23.0 (10.0–40.0)	0.108
C‐reactive protein, median (IQR), mg/L	4.0 (1.2–10.6)	4.8 (1.2–11.8)	3.3 (1.2–8.0)	0.167
Partial pressure of carbon dioxide, median (IQR), mm Hg	52.0 (41.0–69.0)	52.0 (41.0–69.0)	52.0 (42.0–69.0)	0.676
Partial pressure of arterial oxygen, median (IQR), mm Hg	57.0 (48.0–70.0)	58.0 (49.0–70.0)	54.0 (47.0–68.0)	0.259
Tricuspid regurgitation velocity, median (IQR), m/s	3.6 (3.2–4.1)	3.6 (3.2–4.1)	3.7 (3.1–4.1)	0.684
Echocardiographic PASP, median (IQR), mm Hg	63.0 (49.0–77.0)	62.0 (49.0–77.0)	66.0 (49.0–80.0)	0.143
Right ventricular diameter, median (IQR), mm	35.0 (30.0–42.0)	35.0 (30.0–41.0)	36.0 (29.0–43.0)	0.489
Right atrial diameter, median (IQR), mm	42.0 (36.0–49.0)	41.0 (36.0–49.0)	42.0 (36.0–49.0)	0.901
Left atrial diameter, median (IQR), mm	32.0 (28.0–37.0)	32.0 (28.0–36.0)	32.0 (29.0–37.0)	0.218
Pulmonary artery diameter, median (IQR), mm	28.0 (25.0–32.0)	28.0 (24.0–32.0)	28.0 (25.0–33.0)	0.428
Ejection fractions, median (IQR), %	69.0 (64.0–75.0)	70.0 (64.0–76.0)	69.0 (64.0–74.0)	0.580
Mean right atrial pressure, median (IQR), mm Hg	4.0 (2.0–8.0)	4.0 (2.0–8.0)	5.0 (2.0–8.0)	0.718
Mean right ventricular pressure, median (IQR), mm Hg	22.0 (16.0–30.0)	22.0 (16.0–29.0)	22.0 (17.0–31.0)	0.328
Mean pulmonary arterial pressure, median (IQR), mm Hg	35.0 (28.0–45.0)	35.0 (28.0–44.0)	37.0 (28.0–48.0)	0.345
Pulmonary artery wedge pressure, median (IQR), mm Hg	9.0 (6.0–12.0)	9.0 (6.0–12.0)	8.0 (6.0–12.0)	0.850
Cardiac output, median (IQR), L/min	4.9 (4.1–6.1)	4.9 (4.1–6.1)	5.0 (4.0–6.1)	0.526
Cardiac index, median (IQR), L/min/m^2^	3.1 (2.6–3.9)	3.2 (2.6–3.9)	3.1 (2.6–3.6)	0.454
Pulmonary vascular resistance, median (IQR), Wood *U*	5.1 (3.5–8.0)	5.1 (3.5–7.8)	5.4 (3.3–8.9)	0.221
Total pulmonary resistance, median (IQR), Wood *U*	6.9 (5.1–10.1)	6.8 (5.1–10.0)	7.1 (5.4–11.5)	0.222
Systemic vascular resistance, median (IQR), Wood *U*	18.8 (15.0–23.1)	19.0 (15.0–23.0)	18.7 (15.0–23.3)	0.680

Two‐tailed *p* values <0.05 were considered statistically significant.

Abbreviations: COPD, chronic obstructive pulmonary disease; FEV1, forced expiratory volume in 1 s; FEV1/FVC, forced expiratory volume in 1 s/forced vital capacity; GOLD, global initiative for chronic obstructive lung disease; HDL‐C, high‐density lipoprotein cholesterol; IQR, interquartile range; LDL‐C, low‐density lipoprotein cholesterol; NT‐proBNP, N‐terminal pro‐brain natriuretic peptide; NYHA, New York Heart Association; PASP, pulmonary artery systolic pressure; PH, pulmonary hypertension.

### 3.2 Developing and validating the non‐invasive risk assessment nomogram

**FIGURE 1:**
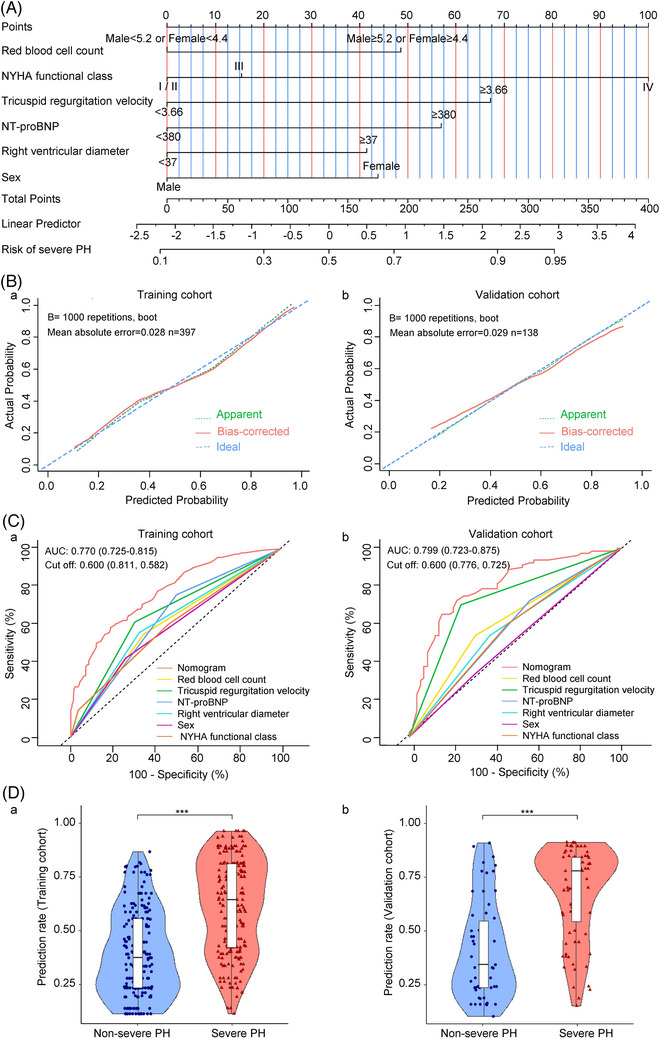
Development and validation of the non‐invasive nomogram. (A) The nomogram incorporates six variables, with points allocated according to the scale for each variable. A total score could be easily calculated by adding each single score, and the total score would then be used to calculate the predicted probability of severe PH in COPD. (B (a, b)) Calibration curves for the non‐invasive nomogram in the training (a) and validation (b) cohorts. The calibration plot illustrates the accuracy of the original prediction (‘Apparent’; light dotted line) and bootstrap models (‘Bias‐corrected’; solid line) in predicting the probability of severe PH in COPD. The 45° straight line represents the perfect match between the actual and nomogram‐predicted probabilities. A closer distance between the two curves indicates higher accuracy. (C (a, b)) ROC curves of the non‐invasive nomogram, red blood cell count, tricuspid regurgitation velocity, NT‐proBNP, right ventricular diameter, sex and NYHA functional class in the training (a) and validation (b) cohorts. Red represents the non‐invasive nomogram, yellow represents red blood cell count, green represents tricuspid regurgitation velocity, dark blue represents NT‐proBNP, blue represents right ventricular diameter, purple represents sex and brown represents NYHA functional class. (D (a, b)) Violin plot analysis comparing the distribution of risk prediction probabilities for non‐severe PH versus severe PH in COPD groups in the training (a) and validation (b) cohorts. The predicted risk probabilities for severe PH groups in both cohorts were much higher than those for non‐severe PH groups. A violin plot and the depicted data are shown. Three lines within the plot show the first and third quartiles and the median of the dataset, whereas the width of the violin body indicates the density of data along the *Y*‐axis. The edges of the violins represent the minimum and maximum values of the dataset. COPD, chronic obstructive pulmonary disease; NT‐proBNP, N‐terminal pro‐brain natriuretic peptide; NYHA, New York Heart Association; PH, pulmonary hypertension.

In the univariate analysis, multiple non‐invasive clinical parameters were associated with severe PH in patients with COPD (Supporting Information Table E2). In the multivariate analysis, TRV, N‐terminal pro‐brain natriuretic peptide (NT‐proBNP), the red blood cell count, New York Heart Association (NYHA) functional class, right ventricular diameter and sex were independent predictors of severe PH in patients with COPD. We calculated the best cut‐off score for these independent predictors according to the Youden Index (Supporting Information Figure E2A). These six independent predictors were used to construct a non‐invasive clinical nomogram for predicting the risk of severe PH in patients with COPD (Figure 1A). The scores for each variable were obtained, and the sum of these scores was recorded as the total score. The prediction risk corresponding to the total score represented the risk of severe PH in patients with COPD (Supporting Information Figure E2B).

The calibration plot showed that the predicted probabilities were close to the actual observed outcomes in the training (Figure 1B,a) and validation cohorts (Figure 1B,b). The Hosmer–Lemeshow test of the training and validation cohort models (*χ* ^2^ = 77.491, *p *= 0.051 and *χ* ^2^ = 24.591, *p *= 0.623, respectively) indicated a good fit of the nomogram. The AUC value of the training and validation cohorts were 0.770 (95% CI, 0.725–0.815; Figure 1C,a) and 0.799 (95% CI, 0.723–0.875; Figure 1C,b), respectively. The compound multivariable nomogram model outperformed the independent factors alone for predicting the risk of severe PH in patients with COPD. Moreover, DCA curves showed that the nomogram might better predict the risk of severe PH in patients with COPD because they added more net benefits than the treat‐all and treat‐none schemes in the training (Supporting Information Figure E2C,a) and validation cohorts (Supporting Information Figure E2C,b). The nomogram constructed using logistic regression was used to calculate the probability of developing severe PH in all patients with COPD. The predicted risks in the severe PH groups were markedly higher than those in the non‐severe PH groups in both cohorts. The nomogram predicted the probability of severe and non‐severe PH as 0.647 (IQR: 0.422–0.817) versus 0.372 (IQR: 0.230–0.559, *p* < 0.001) in the training cohort and 0.795 (IQR: 0.528–0.860) versus 0.354 (IQR: 0.248–0.576, *p* < 0.001) in the validation cohort (Figure 1D,a,b). These findings suggested that the risk assessment prediction model could accurately predict the risk of severe PH in patients with COPD.

### 3.3 Developing and validating the prognostic nomogram

**FIGURE 2:**
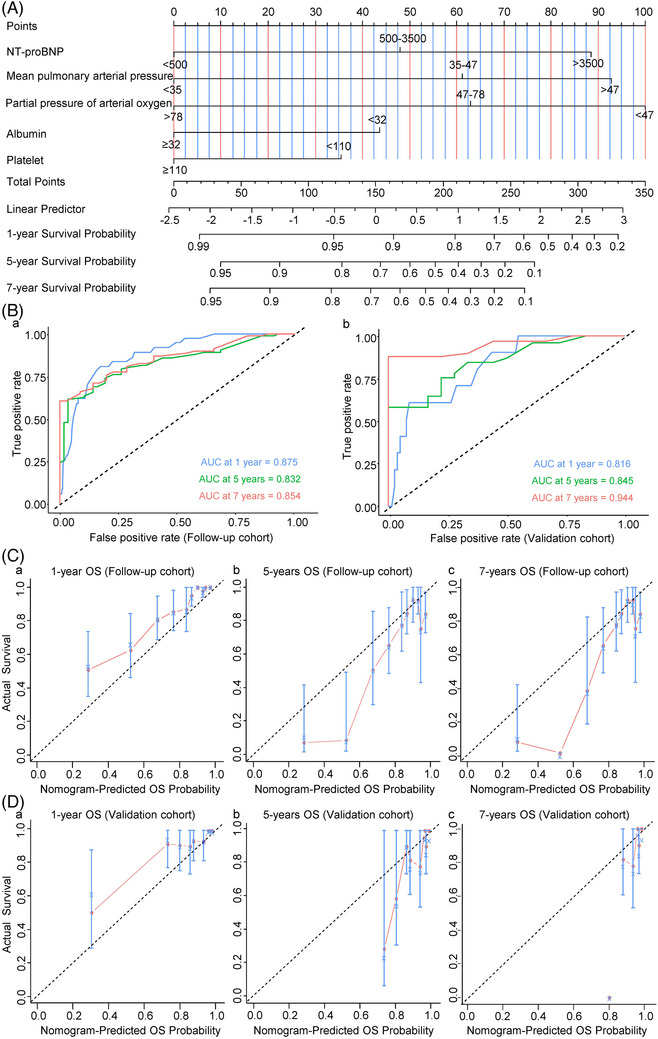
Development and validation of the prognostic nomogram. (A) The nomogram incorporates five variables, with points allocated according to the scale for each variable. A total score could be easily calculated by adding each single score, and the total score was then used to calculate the predicted 1‐, 5‐ and 7‐year OS of COPD–PH. (B (a, b)) ROC curve of the 1‐, 5‐ and 7‐year survival prediction in the follow‐up (a) and validation cohorts (b). (C (a–c)) Calibration curves of the 1‐, 5‐ and 7‐year OS for COPD–PH in the follow‐up cohort. (D (a–c)) Calibration curves of the 1‐, 5‐ and 7‐year OS for COPD–PH in validation cohort. The light blue line indicates the ideal reference line where predicted probabilities would match the observed survival rates. The red dots are calculated by bootstrapping (resample: 1000) and represent the performance of the nomogram. The closer the solid red line is to the light blue line, the more accurately the model predicts survival. AUC, area under the curve; COPD, chronic obstructive pulmonary disease; NT‐proBNP, N‐terminal pro‐brain natriuretic peptide; OS, overall survival; PH, pulmonary hypertension; ROC, receiver operating characteristic.

In the multivariate model of the follow‐up cohort, NT‐proBNP, mPAP, partial pressure of arterial oxygen (PaO_2_), albumin and platelet were identified as independent prognostic factors of COPD–PH (Supporting Information Table E3). The optimal cut‐off values for the independent prognostic factors were identified using the X‐tile plots. NT‐proBNP, mPAP, PaO_2_, albumin and platelet were significantly associated with OS (Supporting Information Figure E3A) for creating an additional nomogram to predict the OS of COPD–PH (Figure 2A). In applying nomograms, each variable can be assigned a value, and the sum of these represents the overall score. The predicted 1‐, 5‐ and 7‐year OS in patients with COPD–PH corresponded to the overall score (Supporting Information Figure E3B).

We then applied the time‐dependent AUC to verify the predictive capacity of the prognostic model. The AUCs for predicting OS of 1, 5 and 7 years in the follow‐up cohort were 0.875, 0.832 and 0.854, respectively (Figure 2B,a), whereas those in the validation cohort were 0.816, 0.845 and 0.944, respectively (Figure 2B,b). The nomogram's calibration curves demonstrated good concordance among the predicted and observed survival rates at 1, 5 and 7 years in the follow‐up cohort (Figure 2C,a–c) and in the validation cohort (Figure 2D,a–c). These results indicated a satisfactory predictive performance.

**FIGURE 3:**
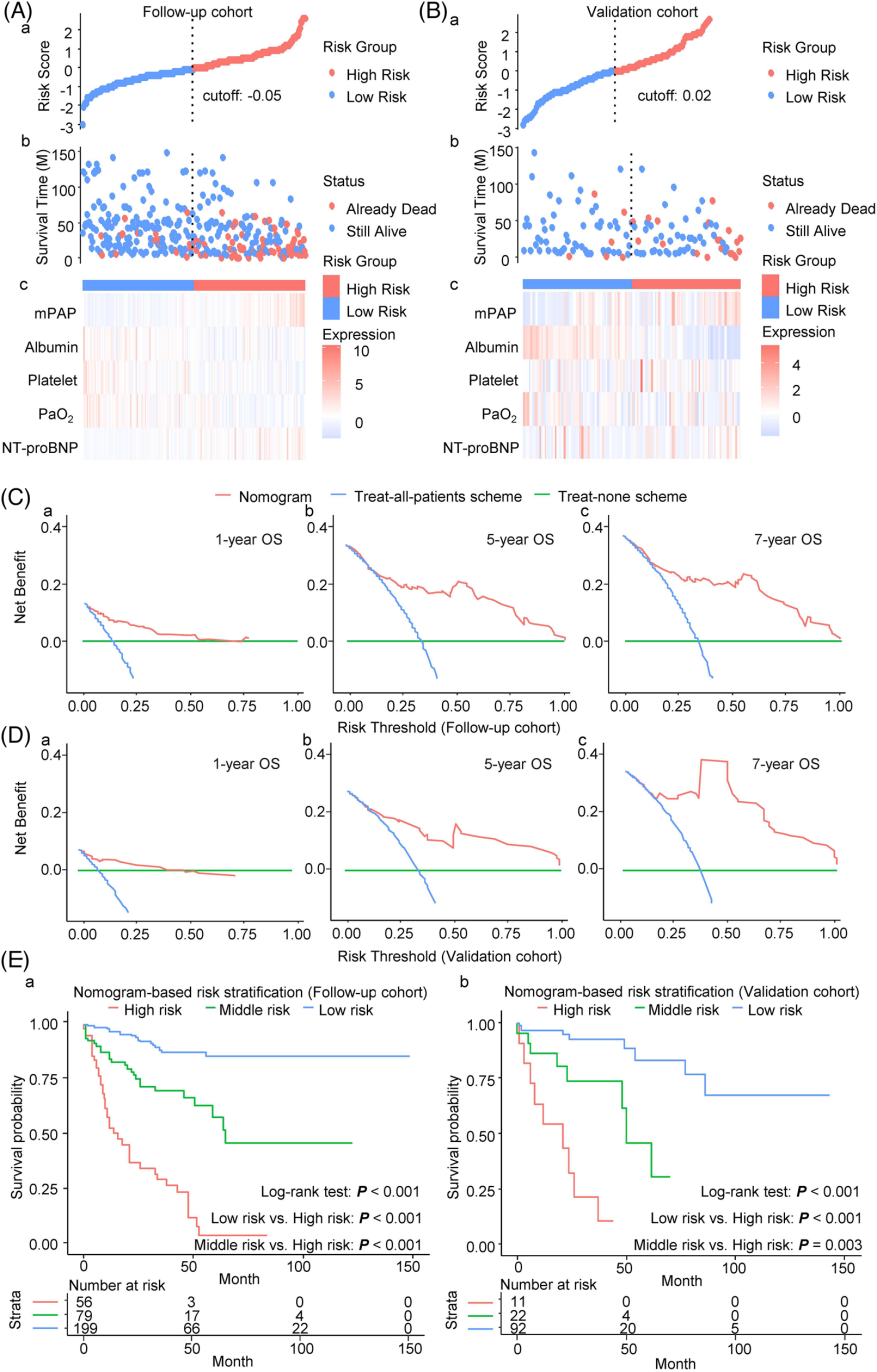
Clinical usefulness of the prognostic nomogram. (A, B) The distribution plot of the risk score in the follow‐up (A) and validation cohorts (B). Patients are arranged from left to right in increasing order of risk score (a). The survival status of each patient (b). The *Y*‐axis represents the overall survival time. The colour code: blue for alive cases and red for dead cases. Heatmap of the expression levels of the five variables (c). (C, D) Decision curve analysis for the prognostic nomogram in the follow‐up (C) and validation cohorts (D). The *Y*‐axis indicates the net benefit, which is calculated by summing the benefits (true positives) and subtracting the harms (false positives). The *X*‐axis indicates the threshold probability. (E (a, b)) Kaplan–Meier overall survival curves for the low‐risk, middle‐risk and high‐risk COPD–PH patients stratified by the prognostic nomogram in the follow‐up (a) and validation cohorts (b). COPD, chronic obstructive pulmonary disease; mPAP, mean pulmonary arterial pressure; M, month; NT‐proBNP, N‐terminal pro‐brain natriuretic peptide; OS, overall survival; PaO_2_, partial pressure of arterial oxygen; PH, pulmonary hypertension.

We also performed a risk stratification based on total scores from the prognostic nomogram. The risk distribution plot and heatmap of each patient with COPD–PH in the follow‐up and validation cohorts are shown in Figure 3A,a–c,B,a–c, respectively. Most patients with COPD–PH in the high‐risk group died, and the variables selected for the prognostic nomogram were closely related to the survival status (Figure 3A,B). This finding further validated the feasibility of the constructed prognostic nomogram. Moreover, the DCA curve showed that the nomogram better predicted OS because the nomogram had more net benefits than the treat‐all and the treat‐none schemes in the follow‐up (Figure 3C,a–c) and validation cohorts (Figure 3D,a–c). Furthermore, the X‐tile plots showed that the optimal threshold values for mortality risk scores were 139.7 and 193.3 (Supporting Information Figure E3C). These values were used to classify patients with COPD–PH into the following three risk groups: low risk (total points <139.7), middle risk (total points ≥139.7 and <193.3) and high risk (total points ≥193.3). The Kaplan–Meier OS curves showed excellent discrimination between the three risk groups. The survival time of the high‐risk group was significantly shorter than that in the middle‐risk and low‐risk groups in the follow‐up (Figure 3E,a, *p* < 0.001) and validation cohorts (Figure 3E,b, *p* < 0.001).

### 3.4 Implementation of the web server

Finally, we designed two online nomograms to allow easy and efficient use in clinical practice. The final non‐invasive model incorporated six independent predictors (NYHA functional class, TRV, right ventricular diameter, sex, the red blood cell count and NT‐proBNP) and was developed as an online tool (https://copd‐copd.shinyapps.io/DynNomapp2/, Supporting Information Figure E4A). The final prognostic prediction model incorporated five independent predictors (NT‐proBNP, mPAP, PaO_2_, albumin and platelet) and was constructed as an online tool (https://copd‐ph.shinyapps.io/DynNomapp/, Supporting Information Figure E4B–D). To predict the risk of severe PH using the web server, the patient's features are shown on the left. To select subgroups from the dropdown menu, the progress bar can be dragged to choose indicator values. After selecting COPD clinical characteristics, users can click ‘Predict’, and the right side generates disease risk prediction and the 95% CI. Users can click ‘Numerical Summary’ and ‘Model Summary’ for data tables and an overview. To use the prognostic prediction nomogram, the clinical features can be entered on the left side. Users can then check ‘Predicted Survival at this Follow Up’, select the prediction time and click the ‘Predict’ button, and a ‘Survival plot’ will be generated on the right side. Clicking on ‘Predicted Survival’ will obtain the predicted survival probability and 95% CI. Clicking on ‘Numerical summary’ and ‘Model summary’ will enable viewing of the data table and an overview of the nomogram.

## 4 DISCUSSION

This study specifically focused on using multicentre data and the latest European Society of Cardiology guidelines to develop and validate a non‐invasive nomogram. We aimed to predict the risk of severe PH in patients with COPD and conduct a long‐term follow‐up study on the prognosis of COPD–PH, with external validation using multicentre datasets. Furthermore, we developed an online tool with a digital interface (https://copd‐copd.shinyapps.io/DynNomapp2/), facilitating early identification of severe PH in patients with COPD and better clinical decision making. We also developed another online nomogram (https://copd‐ph.shinyapps.io/DynNomapp/) to predict the OS of patients with COPD–PH and monitor the situation.

Consistent with a recent study,^18^ this study showed that NT‐proBNP was not only an independent factor for predicting severe PH in patients with COPD,^18^ but also an independent prognostic factor in patients with COPD–PH. These findings further indicated that elevated plasma NT‐proBNP could serve as a biomarker for monitoring the progression of COPD and identifying secondary PH. Furthermore, the pulmonary function data in our study have a limitation in the risk assessment of severe PH in patients with COPD and the prognosis of COPD–PH. This finding is consistent with recent studies,^19^, ^20^ which showed that airflow obstruction ([FEV1/FVC] ratio) could not predict death because PH had little effect on lung mechanics. Moreover, PaO_2_ was a prognostic factor in patients with COPD–PH in our study, which further emphasises the independent prognostic value of hypoxemia in COPD–PH^21^ and provides evidence that long‐term oxygen therapy can slow the progression of COPD–PH.^22^ Our findings suggested that when the pulmonary function of patients with COPD was relatively preserved and PaO_2_ was low, clinicians should suspect out‐of‐proportion PH, which was defined as mPAP >35–40 mm Hg and a mild‐to‐moderate airflow limitation.^20^ This finding has important implications for the diagnosis and treatment of patients with COPD.

Notably, we found that females with COPD were more likely to develop severe PH than males with COPD, suggesting a susceptibility of females to severe PH. Surprisingly, females with severe PH were younger and had better survival than males with severe PH. This finding suggests that females may have some advantage in coping with PH. Differences in genetics, hormonal regulation and immune responses between females and males may affect the development and progression of PH. Sex may play a role in COPD–PH, but further research is required to understand the reasons for these differences.

Compared to relying solely on PVR measured by RHC for grading the severity of COPD–PH in patients,^11^ the nomogram established in this study provides a non‐invasive disease management tool with good predictive efficacy for such patients. The development of online risk assessment nomogram software will provide a clinical tool for long‐term management of patients with COPD and COPD–PH in primary hospitals or community centres. In addition, in contrast to previous single‐centre studies,^18^, ^23^, ^24^ our study included a larger sample of patients with COPD–PH, and used a multicentre study design and internal and external validation. These processes contributed to improving the reliability and generalisability of our research findings. Unlike previous studies,^18^, ^23^, ^24^ this study adheres to the latest diagnostic criteria from the European Society of Cardiology guidelines. Moreover, we used RHC to show PH in patients with COPD, which is more accurate than other studies that used Doppler echocardiography to diagnose PH.^23^, ^24^ Our risk assessment nomogram may have clinical implications for individualised follow‐up and guidance of therapeutic strategy in patients. Optimisation of such software will lessen the requirement for the clinician's subjective impression or time‐consuming manual measurements. By integrating online servers into hospital systems, doctors can better understand patients’ conditions. An online network provides doctors with artificial intelligence‐assisted diagnostic and decision‐making capabilities to facilitate an accurate diagnosis, early treatment, and personalised strategies. Moreover, our study introduces a risk assessment tool specifically designed for Chinese patients with COPD–PH, and provides a new perspective on risk stratification management for these patients.

Although these nomograms performed well, this study has some limitations. First, this retrospective study had potential selection bias. The indication for RHC in patients with COPD was a TRV >2.8 m/s,^11^ which may indicate PH. The predictive model may be suitable for risk stratification and prognosis of patients who are highly suspected of having COPD–PH. Second, because of unavailability of data in the datasets, certain potential predictors (e.g., carbon monoxide diffusing capacity, tricuspid annular plane systolic excursion, right ventricular fractional area change and tissue Doppler imaging) were not incorporated into the multivariable model. Third, larger samples, multicentre, prospective and randomised controlled trials are still required for validation and optimisation of our results.

In summary, the proposed nomograms could be used as non‐invasive clinical tools to enhance the risk assessment of severe PH in patients with COPD and prognosis of COPD–PH. Additionally, the online network software that we developed may provide a clinical tool for long‐term management of patients with COPD and those with COPD–PH in primary hospitals or community centres. This software has the prospect of artificial intelligence‐assisted diagnosis and research and development of treatment.

## AUTHOR CONTRIBUTIONS

Dansha Zhou, Yunshan Cao, Jian Wang, and Yuqin Chen planned, designed research, and revised articles. Yunshan Cao, Jian Wang, Yuqin Chen, Dansha Zhou, Chunli Liu, Lan Wang, JiFeng Li, Yating Zhao, Zheng Deng, and Chi Hou analyzed and tracked data and revised manuscripts. Yingyun Fu, Qian Jiang, Ning Lai, Rui Zhang, Weici Feng, Chuhui Gao, Xiang Li, Mei Jiang, Xin Fu, Jiyuan Chen, Wei Hong, Lei Xu, Wenjun He, Jinming Liu, YuanHua Yang, Wenju Lu, and Nanshan Zhong collected and summarised data. All authors rigorously reviewed the manuscript, provided important intellectual input, approved the final version, and agreed to take responsibility for their contributions. All authors read and approved the final manuscript.

## ETHICS STATEMENT

The study protocol was approved by the Medical Ethics Committee of the First Affiliated Hospital of Guangzhou Medical University (Medical Research Ethics Review No. 09, 2021).

## CONFLICTS OF INTEREST STATEMENT

The authors declare no conflicts of interest.

## Supporting information

Supporting Information

## Data Availability

All data included in this study are available upon request through contact with the corresponding author.
