# Supplementary material for: Prediction of clinical risk assessment and survival in chronic obstructive pulmonary disease with pulmonary hypertension
Source: Clin Transl Med. 2024 Jun 11;14(6):e1702. doi: 10.1002/ctm2.1702 (PMC11166097; doi:10.1002/ctm2.1702)
Supplement: Supplementary file 1 — Supporting Information [file CTM2-14-e1702-s001.docx]

**Supplemental Online Content**

**Prediction of clinical risk assessment and survival in chronic obstructive pulmonary disease with pulmonary hypertension**

**Appendix.** Data collection and statistical analysis

**Figure E1.** Flow chart of the study

**Figure E2.** Forest plot, interactive nomogram, and decision curve analysis for predicting severe PH in COPD

**Figure E3.** Forest plot and interactive nomogram for predicting the overall survival rates of COPD-PH

**Figure E4.** Online Web software in training and follow-up cohorts

**Table E1.** Demographic and clinical characteristics of patients with COPD-PH in follow-up and validation cohorts

**Table E2.** Selected factors in the training cohort for building the prediction model by univariate and multivariate logistics regression analysis

**Table E3.** Selected factors in the follow-up cohort for building the prognostic model by univariate and multivariate Cox regression analysis

**Appendix.** Data collection and statistical analysis

**Data collection**

Demographic and clinical characteristics, pulmonary function tests, biochemical tests, and hemodynamic data (echocardiography) were collected to construct a non-invasive nomogram to predict severe PH in COPD. These variables and hemodynamic data (Right heart catheterization and echocardiography) were incorporated to predict the overall survival of patients with COPD-PH. The data used in this study were collected from The First Affiliated Hospital of Guangzhou Medical University, Shanghai Pulmonary Hospital, Gansu Provincial Hospital, Beijing Chao-Yang Hospital, The First People’s Hospital of Yunnan, and Shenzhen People’s Hospital. A senior respiratory physician from each institution supervised the data collection process from the patients’ registries and electronic medical records, as well as the patients’ follow-up. The smoking status (never-smoker, ex-smoker, and current smoker) was based on the classification of the United States Center for Disease Control and Prevention.

**Statistical analysis**

We divided the patients with COPD-PH into high-risk, middle risk, and low-risk groups using the prognostic nomogram to calculate the patients’ scores in relation to mortality. The optimal risk score for the mortality cut-off value in the follow-up cohort was identified using X-tile plots ^1^. A Kaplan–Meier survival analysis was used to examine a significant difference in patients’ survival with the log-rank test. If there were missing data (1%–15%), the multiple imputation method was selected to interpolate the missing values, and variables with a high frequency of missing values were mainly excluded from the multivariate model ^2^.

All statistical analyses were performed using the SPSS statistical software (version 26.0; IBM, Armonk, NY, USA), and R software (version 4.0.2; R Foundation for Statistical Computing, Vienna, Austria).

**References**

1. Camp RL, Dolled-Filhart M, Rimm DL. X-tile: a new bio-informatics tool for biomarker assessment and outcome-based cut-point optimization. Clin Cancer Res. Nov 1 2004;10(21):7252-9. doi:10.1158/1078-0432.Ccr-04-0713

2. Blazek K, van Zwieten A, Saglimbene V, Teixeira-Pinto A. A practical guide to multiple imputation of missing data in nephrology. Kidney international. Jan 2021;99(1):68-74. doi:10.1016/j.kint.2020.07.035

**SUPPLEMENTARY FIGURES**

**
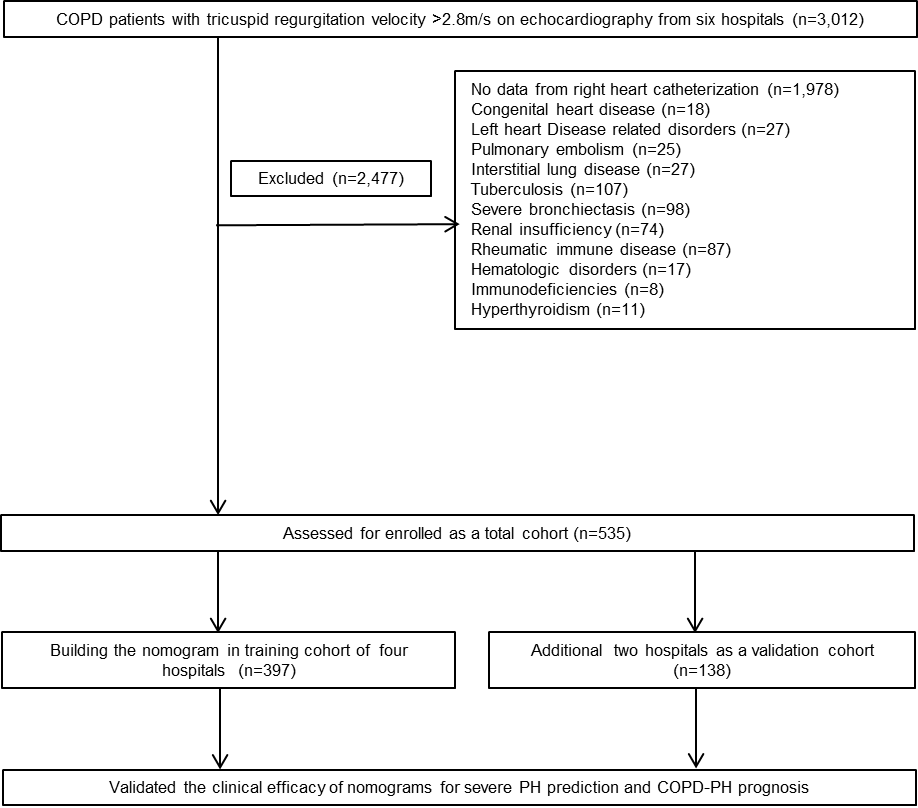
**

**Figure E1. Flow chart of the study**

A total of 535 patients with COPD-PH who met the study’s eligibility criteria between December 2008 and July 2021 were chosen from six Chinese hospitals to construct and validate a non-invasive diagnostic nomogram for predicting severe PH in COPD and a prognostic nomogram for predicting the overall survival rates of COPD-PH. COPD, chronic obstructive pulmonary disease; RHC, right heart catheterization; PH, pulmonary hypertension.

**
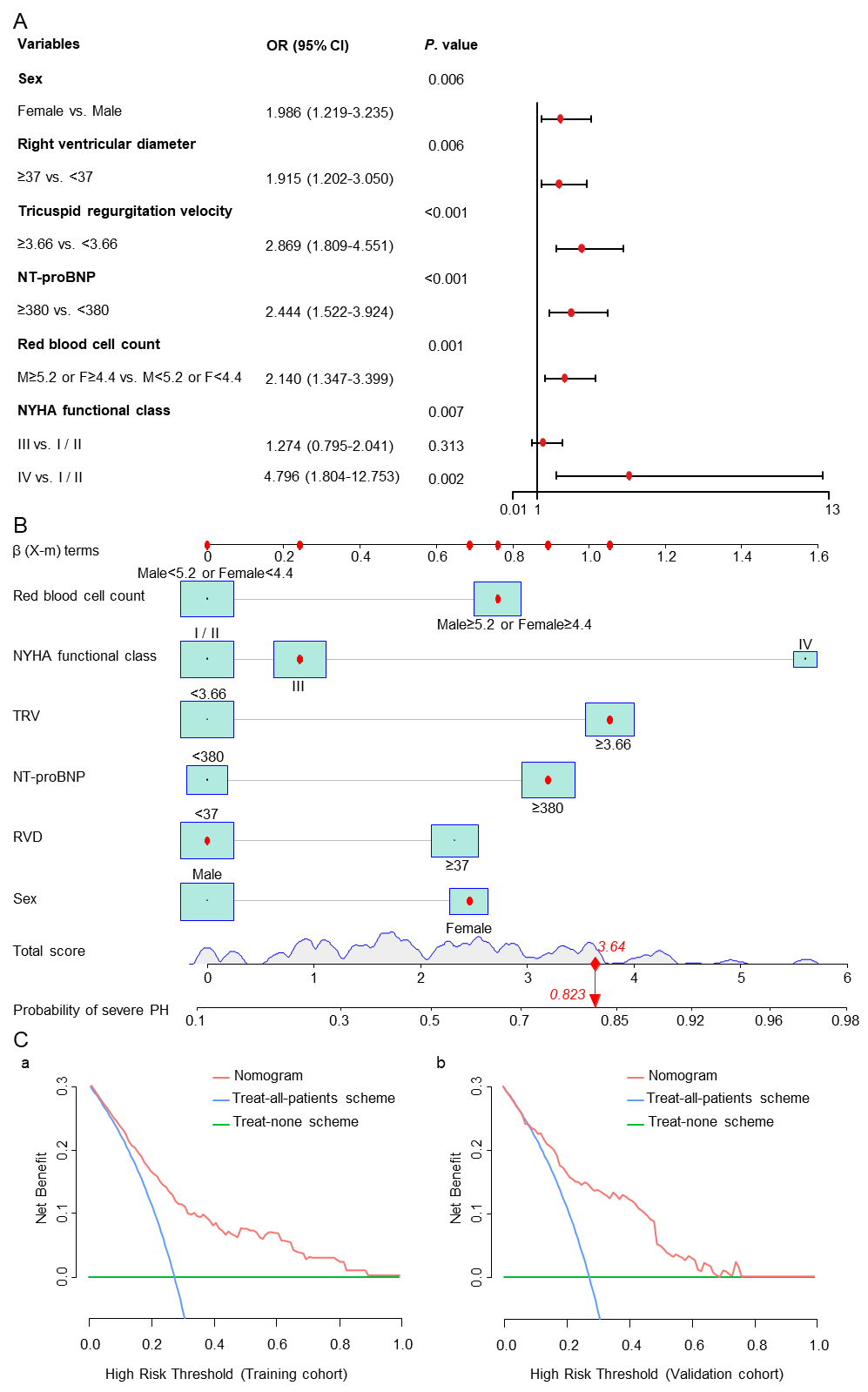
**

**Figure E2. Forest plot, interactive nomogram, and decision curve analysis for predicting severe PH in COPD**

***A:*** Forest plot showing multivariate logistic regression analysis of the potential risk of severe PH in COPD. ***B:*** A female COPD patient with right ventricular diameter <37 mm, NT-proBNP $\geq$380 ng/L, tricuspid regurgitation velocity $\geq$3.66 m/s, NYHA class III, and red blood cell count $\geq$4.4*10^12/L. The probability of severe PH in this patient was 0.823 in this diagnostic nomogram. ***C (a-b):*** Decision curve analysis for the diagnostic nomogram in the training (a) and validation (b) cohorts. The Y-axis indicates the net benefit, which is calculated by summing the benefits (true positives) and subtracting the harms (false positives). The X-axis indicates the threshold probability. CI, confidence interval; COPD, chronic obstructive pulmonary disease; F, female; M, male; NT-proBNP, N-terminal pro-brain natriuretic peptide; NYHA, New York Heart Association; OR, odd ratio; PH, pulmonary hypertension; RVD, right ventricular diameter; TRV, tricuspid regurgitation velocity.

**
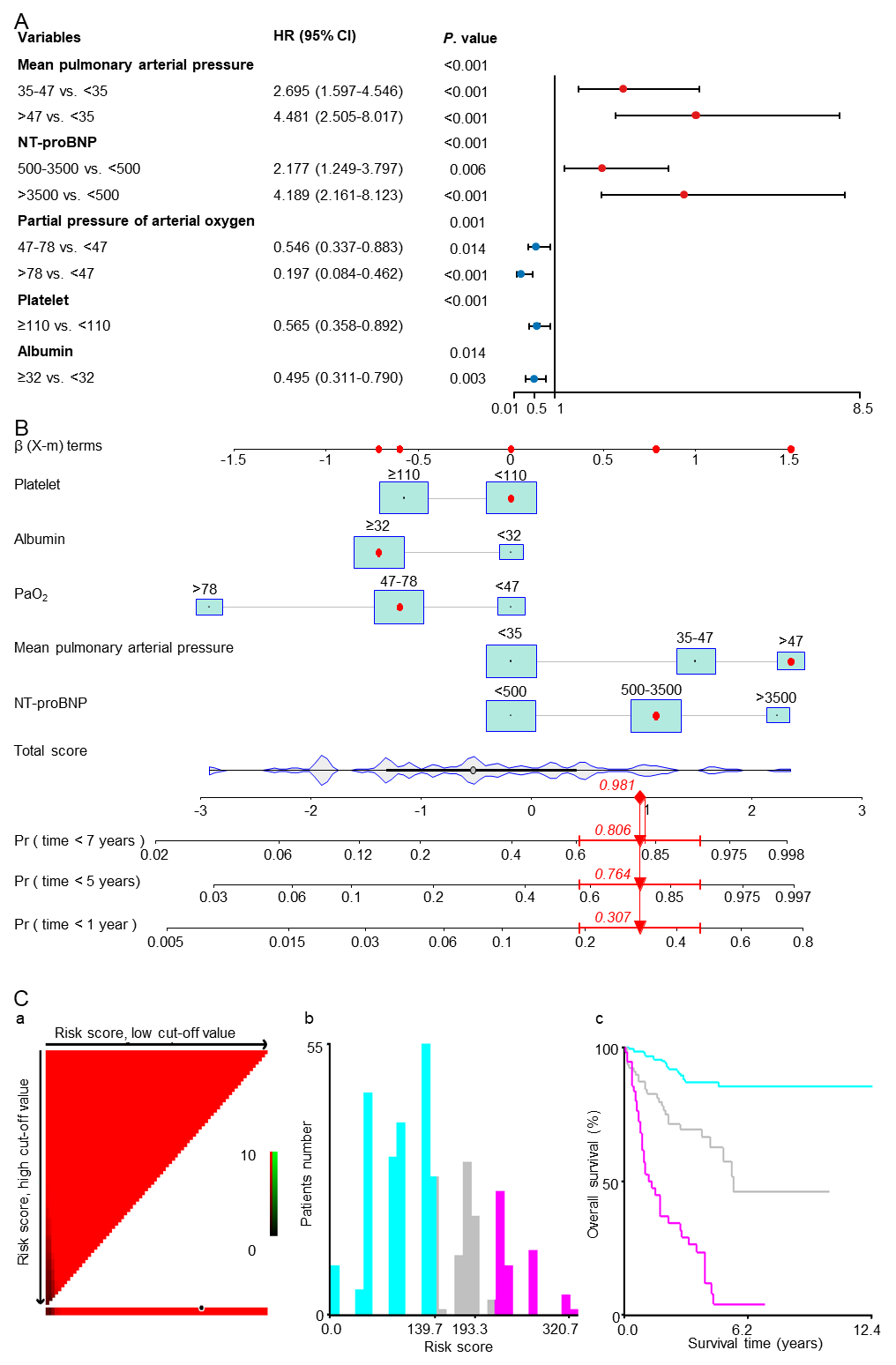
**

**Figure E3. Forest plot and interactive nomogram for predicting the overall survival rates of COPD-PH**

***A:*** Forest plot showing multivariate Cox regression analysis of the potential risk of COPD-PH. ***B:*** COPD-PH patients with NT-proBNP of 500-3500 ng/L, partial pressure of arterial oxygen of 47-78 mmHg, albumin $\geq$32 g/L, platelet <110*10^9/L, and mean pulmonary arterial pressure >47 mmHg confirmed by right heart catheterization. The predicted 1-year, 5-year, and 7-year mortality rates were 0.307, 0.764, and 0.806, respectively, in this prognostic nomogram.

***C (a-b):*** X-tile plots to identify the optimal risk score cut-off based on OS in the follow-up cohort. X-tile plot for the follow-up cohort (a). The X-tile plot was generated by dividing risk scores into three risk groups (low, middle, and high). Each pixel (point) of the X-tile plot represents the data from a given set of partitions. The X-axis represents all potential risk score cut-off values that define a low subset are from low to high (left to right), while the Y-axis means that the risk score cut-off values that define a high subset from high to low (top to bottom). The arrows represent the direction in which the size of the lower subset (X-axis) and the higher subset (Y-axis) increases. The data along the hypotenuse represent the result from a single cut-off value that divides the data into a high or low subset. The color of the plot represents the strength of the association for each partition, ranging from low (dark, black) to high (bright, red, or green). The negative correlation between risk score and survival is shown in red, while the direct correlation is shown in green. The distributions of the number of patients with COPD-PH by risk score (b). Kaplan‒Meier plots categorized by the low-risk, middle-risk, and high-risk groups according to the optimal risk score cut-off. The optimal OS risk score cut-off values were 139.7 and 193.3 (χ^2^ = 140.65, *P* < 0.001) (c). COPD, chronic obstructive pulmonary disease; CI, confidence interval; HR, hazard ratio; NT-proBNP, N-terminal pro-brain natriuretic peptide; PaO_2_, partial pressure of arterial oxygen; PH, pulmonary hypertension; Pr, probability.


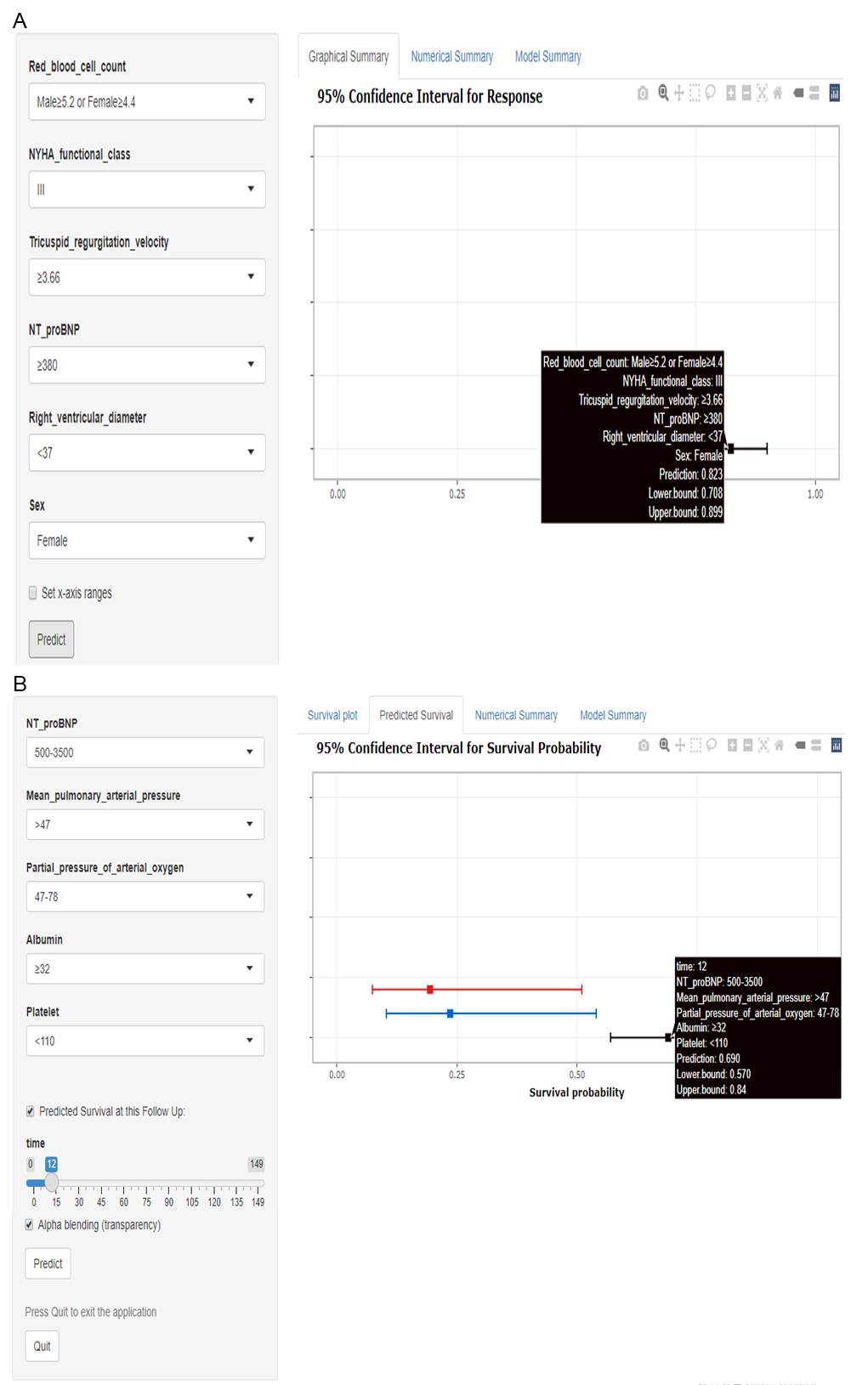


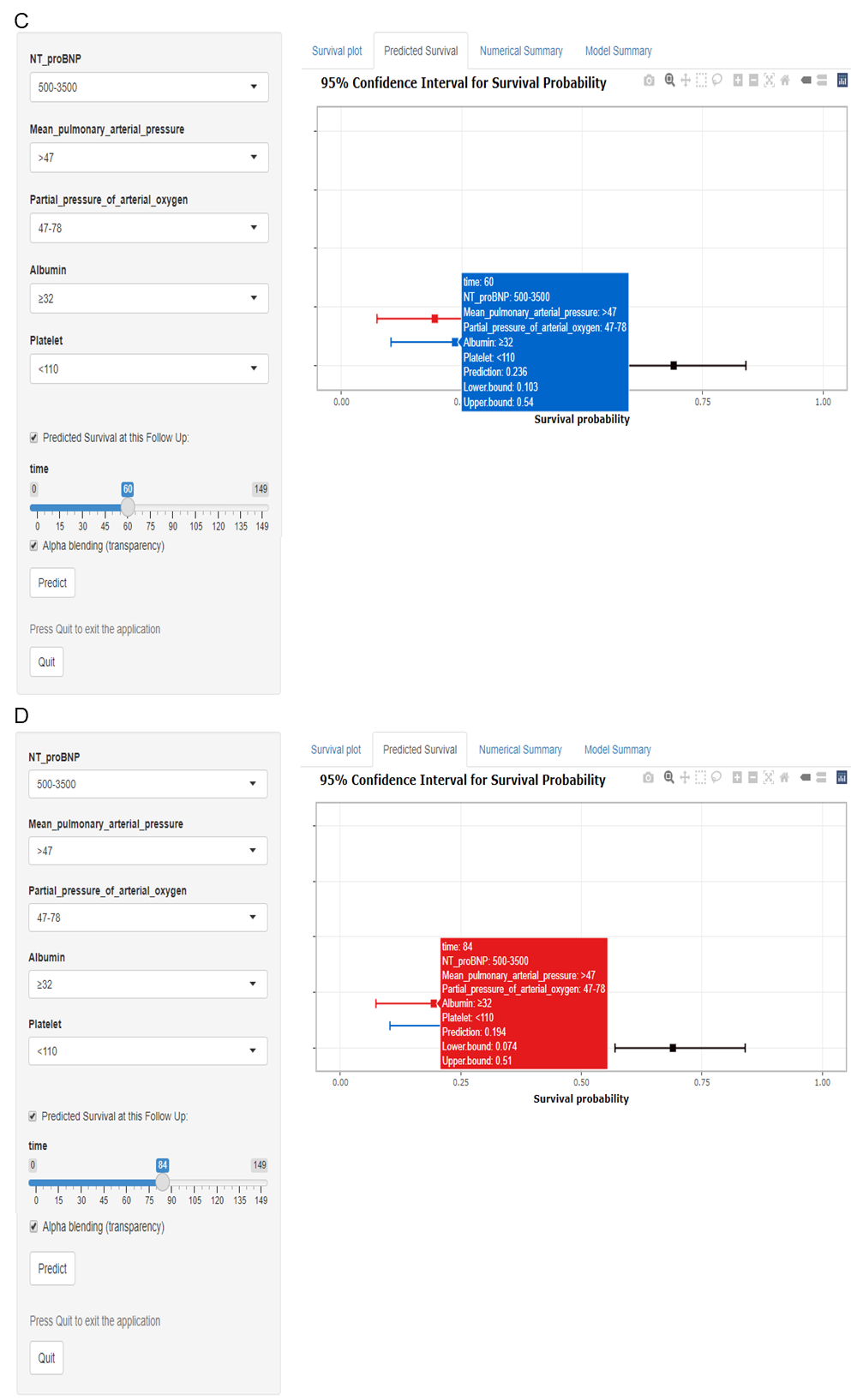


**Figure E4. Online Web software in training and follow-up cohorts**

***A:*** Online nomogram accessible at <https://copd-copd.shinyapps.io/DynNomapp2/>, predicting the risk of severe PH in COPD. A female COPD patient with right ventricular diameter <37 mm, NT-proBNP $\geq$380 ng/L, tricuspid regurgitation velocity $\geq$3.66 m/s, NYHA class III, and red blood cell count $\geq$4.4*10^12/L. The online diagnostic nomogram showed that the probability of this patient suffering from severe PH was 0.823 (95% CI, 0.708-0.899). ***B-D:*** Online nomogram accessible at <https://copd-ph.shinyapps.io/DynNomapp/>, predicting the survival rates for COPD-PH. COPD-PH patients with NT-proBNP of 500-3500 ng/L, partial pressure of arterial oxygen of 47-78 mmHg, albumin $\geq$32 g/L, platelet <110*10^9/L, and mean pulmonary arterial pressure >47 mm Hg showed 1-year, 5-year, and 7-year survival rates were 0.690 (95% CI, 0.570-0.840), 0.236 (95% CI, 0.103-0.540), and 0.194 (95% CI, 0.074-0.510), respectively, in the online prognostic nomogram. COPD, chronic obstructive pulmonary disease; CI, confidence interval; NT-proBNP, N-terminal pro-brain natriuretic peptide; NYHA, New York Heart Association; PH, pulmonary hypertension.

**SUPPLEMENTARY TABLES**

**Table E1. Demographic and clinical characteristics of patients with COPD-PH in follow-up and validation cohorts**

| **Variable** | **Whole population**  **(n = 459)** | **Follow-up cohort**  **(n = 334)** | **Validation cohort**  **(n = 125)** | ***P* Value** | |
| --- | --- | --- | --- | --- | --- |
| Sex, No. (%) |  |  |  | | 0.893 |
| Female | 152 (33.1) | 110 (32.9) | 42 (33.6) | |  |
| Male | 307 (66.9) | 224 (67.1) | 83 (66.4) | |  |
| Smoking status, No. (%) |  |  |  | | 0.647 |
| Non-smoker | 265 (57.7) | 191 (57.2) | 74 (59.2) | |  |
| Ex-smoker | 52 (11.3) | 36 (10.8) | 16 (12.8) | |  |
| Smoker | 142 (30.9) | 107 (32.0) | 35 (28.0) | |  |
| Severity of PH, No. (%) |  |  |  | | 0.505 |
| Non-severe PH | 232 (50.5) | 172 (51.5) | 60 (48.0) | |  |
| Severe PH | 227 (49.5) | 162 (48.5) | 65 (52.0) | |  |
| NYHA functional class, No. (%) |  |  |  | | 0.017 |
| I / II | 207 (45.1) | 164 (49.1) | 43 (34.4) | |  |
| III | 210 (45.8) | 143 (42.8) | 67 (53.6) | |  |
| IV | 42 (9.2) | 27 (8.1) | 15 (12.0) | |  |
| GOLD stage, No. (%) |  |  |  | | 0.855 |
| Stage I | 24 (5.2) | 19 (5.7) | 5 (4.0) | |  |
| Stage II | 103 (22.4) | 75 (22.5) | 28 (22.4) | |  |
| Stage III | 184 (40.1) | 135 (40.4) | 49 (39.2) | |  |
| Stage IV | 148 (32.2) | 105 (31.4) | 43 (34.4) | |  |
| Survival outcome, No. (%) |  |  |  | | 0.272 |
| Alive | 347 (75.6) | 248 (74.3) | 99 (79.2) | |  |
| Dead | 112 (24.4) | 86 (25.7) | 26 (20.8) | |  |
| Follow-up duration, median (IQR), month | 28.0 (11.0-49.0) | 31.0 (12.0-51.0) | 23.0 (9.0-45.0) | | 0.028 |
| Age, median (IQR), year | 65.0 (57.0-71.0) | 65.0 (58.0-71.0) | 64.0 (57.0-71.0) | | 0.633 |
| Smoking exposure, median (IQR), year | 0.0 (0.0-30.0) | 0.0 (0.0-30.0) | 0.0 (0.0-30.0) | | 0.694 |
| NT-proBNP, median (IQR), ng/L | 746.0 (203.0-1928.0) | 741.0 (193.0-1897.0) | 782.0 (207.0-2447.0) | | 0.476 |
| Six-Minute Walk Test, median (IQR), meter | 333.0 (197.0-430.0) | 348.0 (202.0-438.0) | 307.0 (157.0-394.0) | | 0.018 |
| FEV1, median (IQR), % | 38.0 (27.0-53.0) | 37.0 (27.0-54.0) | 38.0 (25.0-51.0) | | 0.323 |
| FEV1/FVC, median (IQR), % | 55.0 (46.0-63.0) | 55.0 (46.0-62.0) | 55.0 (45.0-63.0) | | 0.792 |
| Body Mass Index, median (IQR), kg/m^2^ | 21.5 (18.4-24.1) | 21.5 (18.4-24.2) | 21.7 (18.4-23.7) | | 0.461 |
| Red blood cell count, median (IQR), * 10^12/L | 4.9 (4.2-5.5) | 4.9 (4.1-5.5) | 4.9 (4.3-5.5) | | 0.445 |
| White blood cell count, median (IQR), * 10^9/L | 6.3 (5.0-8.4) | 6.3 (5.1-8.4) | 6.4 (4.7-8.3) | | 0.243 |
| Hemoglobin, median (IQR), g/L | 142.0 (126.0-161.0) | 141.0 (124.0-161.0) | 143.0 (127.0-164.0) | | 0.351 |
| Platelet, median (IQR), * 10^9/L | 182.0 (143.0-226.0) | 183.0 (146.0-229.0) | 177.0 (141.0-215.0) | | 0.154 |
| Aspartate Transaminase, median (IQR), U/L | 22.0 (18.0-29.0) | 22.0 (17.0-29.0) | 23.0 (19.0-28.0) | | 0.618 |
| Alanine aminotransferase, median (IQR), U/L | 20.0 (13.0-29.0) | 20.0 (14.0-28.0) | 22.0 (13.0-31.0) | | 0.119 |
| Total bilirubin, median (IQR), μmol/L | 15.0 (10.8-22.5) | 15.0 (11.0-22.2) | 15.0 (11.0-23.2) | | 0.801 |
| Direct Bilirubin, median (IQR), μmol/L | 5.5 (3.3-9.4) | 5.5 (3.4-9.1) | 5.4 (3.0-9.5) | | 0.944 |
| Total protein, median (IQR), g/L | 65.0 (60.0-71.0) | 66.0 (60.0-72.0) | 64.0 (60.0-69.0) | | 0.172 |
| Albumin, median (IQR), g/L | 37.0 (34.0-40.0) | 37.0 (34.0-40.0) | 37.0 (34.0-40.0) | | 0.354 |
| Creatinine, median (IQR), μmol/L | 70.0 (55.0-87.0) | 71.0 (55.0-88.0) | 70.0 (54.0-86.0) | | 0.406 |
| Blood urea nitrogen, median (IQR), mmol/L | 6.3 (4.8-8.6) | 6.4 (4.7-8.9) | 6.0 (4.9-8.0) | | 0.429 |
| D-Dimer, median (IQR), mg/L | 656.0 (320.0-1104.0) | 644.0 (325.0-1131.0) | 668.0 (311.0-1041.0) | | 0.873 |
| Total cholesterol, median (IQR), mmol/L | 4.0 (3.5-5.0) | 4.0 (3.5-5.0) | 4.0 (3.2-5.0) | | 0.042 |
| Triglyceride, median (IQR), mmol/L | 1.0 (0.8-1.8) | 1.0 (0.8-1.8) | 1.0 (0.8-1.5) | | 0.335 |
| HDL-C, median (IQR), mmol/L | 1.1 (1.0-1.5) | 1.1 (1.0-1.5) | 1.1 (1.0-1.4) | | 0.627 |
| LDL-C, median (IQR), mmol/L | 2.5 (2.0-3.0) | 2.4 (2.0-3.0) | 2.6 (2.0-3.0) | | 0.601 |
| Erythrocyte sedimentation rate, median (IQR), mm/h | 30.0 (11.0-48.0) | 31.0 (11.0-50.0) | 24.0 (11.0-41.0) | | 0.240 |
| C-reactive protein, median (IQR), mg/L | 4.3 (1.4-10.7) | 5.0 (1.4-11.8) | 3.5 (1.3-8.0) | | 0.248 |
| Partial pressure of carbon dioxide, median (IQR), mmHg | 52.0 (41.0-70.0) | 52.0 (41.0-69.0) | 52.0 (41.0-71.0) | | 0.469 |
| Partial pressure of arterial oxygen, median (IQR), mmHg | 58.0 (49.0-69.0) | 59.0 (50.0-71.0) | 54.0 (47.0-68.0) | | 0.048 |
| Tricuspid regurgitation velocity, median (IQR), m/s | 3.6 (3.1-4.1) | 3.6 (3.1-4.1) | 3.6 (3.1-4.1) | | 0.582 |
| Echocardiographic PASP, median (IQR), mm Hg | 62.0 (48.0-77.0) | 61.0 (48.0-77.0) | 65.0 (48.0-77.0) | | 0.240 |
| Right ventricular diameter, median (IQR), mm | 35.0 (30.0-41.0) | 35.0 (31.0-41.0) | 36.0 (28.0-43.0) | | 0.780 |
| Right atrial diameter, median (IQR), mm | 42.0 (36.0-49.0) | 41.0 (36.0-49.0) | 42.0 (36.0-48.0) | | 0.967 |
| Left atrial diameter, median (IQR), mm | 32.0 (28.0-37.0) | 32.0 (28.0-37.0) | 32.0 (29.0-37.0) | | 0.469 |
| Pulmonary artery diameter, median (IQR), mm | 28.0 (22.0-29.0) | 28.0 (24.0-32.0) | 28.0 (25.0-33.0) | | 0.530 |
| Ejection Fractions, median (IQR), % | 69.0 (64.0-75.0) | 69.0 (64.0-75.0) | 69.0 (63.0-75.0) | | 0.566 |
| Mean right atrial pressure, median (IQR), mm Hg | 5.0 (2.0-8.0) | 4.0 (2.0-8.0) | 5.0 (2.0-8.0) | | 0.589 |
| Mean right ventricular pressure, median (IQR), mm Hg | 22.0 (16.0-30.0) | 23.0 (16.0-30.0) | 21.0 (16.0-30.0) | | 0.414 |
| Mean pulmonary arterial pressure, median (IQR), mm Hg | 35.0 (28.0-44.0) | 35.0 (28.0-43.0) | 35.0 (28.0-48.0) | | 0.633 |
| Pulmonary artery wedge pressure, median (IQR), mm Hg | 9.0 (6.0-12.0) | 9.0 (6.0-12.0) | 9.0 (6.0-12.0) | | 0.958 |
| Cardiac output, median (IQR), L/min | 5.0 (4.1-6.1) | 5.0 (4.1-6.1) | 5.0 (4.1-6.0) | | 0.654 |
| Cardiac index, median (IQR), L/min/m^2^ | 3.1 (2.6-3.9) | 3.2 (2.6-3.9) | 3.1 (2.6-3.6) | | 0.721 |
| Pulmonary vascular resistance, median (IQR), Wood U | 5.0 (3.3-7.7) | 5.0 (3.5-7.4) | 5.2 (3.1-8.6) | | 0.344 |
| Total pulmonary resistance, median (IQR), Wood U | 6.9 (5.1-10.1) | 6.8 (5.0-9.9) | 7.0 (5.2-11.4) | | 0.421 |
| Systemic vascular resistance, median (IQR), Wood U | 18.5 (15.0-23.0) | 18.5 (14.9-22.7) | 18.6 (15.2-23.1) | | 0.680 |

Two-tailed *P* values < 0.05 were considered statistically significant.

Abbreviations: FEV1, forced expiratory volume in 1 s; FEV1/FVC%, forced expiratory volume in 1 s/forced volume vital capacity ratio; GOLD, global initiative for chronic obstructive lung disease; HDL-C, high-density lipoprotein cholesterol; IQR, interquartile range; LDL-C, low-density lipoprotein cholesterol; NT-proBNP, N-terminal pro-brain natriuretic peptide; NYHA, New York Heart Association; PASP, pulmonary artery systolic pressure; PH, pulmonary hypertension.

**Table E2.** **Selected factors in the training cohort for building the prediction model by univariate and multivariate logistics regression analysis**

| **Variable** | **Univariate analysis** | | **Multivariate analysis** | |
| --- | --- | --- | --- | --- |
|  | **OR (95% CI)** | ***P* value** | **OR (95% CI)** | ***P* value** |
| Sex, No. (%) |  | 0.001 |  | <0.001 |
| Female vs. Male | 2.041 (1.335-3.121) |  | 2.696 (1.624-4.475) |  |
| Smoking status, No. (%) |  | 0.123 |  |  |
| Ex-smoker vs. Non-smoker | 0.864 (0.460-1.622) | 0.649 |  |  |
| Smoker vs. Non-smoker | 0.632 (0.408-0.981) | 0.041 |  |  |
| NYHA functional class, No. (%) |  | 0.001 |  | 0.002 |
| III vs. I / II | 1.407 (0.929-2.130) | 0.106 | 1.509 (0.937-2.432) | 0.091 |
| IV vs. I / II | 5.386 (2.244-12.924) | <0.001 | 5.802 (2.138-15.742) | 0.001 |
| GOLD stage, No. (%) |  | 0.956 |  |  |
| Stage II vs. Stage I | 0.844 (0.377-1.887) | 0.679 |  |  |
| Stage III vs. Stage I | 0.809 (0.374-1.747) | 0.589 |  |  |
| Stage IV vs. Stage I | 0.859 (0.393-1.877) | 0.703 |  |  |
| Age, median (IQR), year | 0.943 (0.920-1.001) | 0.050 |  |  |
| Smoking exposure, median (IQR), year | 0.992 (0.981-1.003) | 0.164 |  |  |
| NT-proBNP, median (IQR), ng/L | 1.000 (1.000-1.000) | <0.001 | 1.000 (1.000-1.000) | 0.009 |
| Six-Minute Walk Test, median (IQR), meter | 1.000 (0.999-1.002) | 0.418 |  |  |
| FEV1, median (IQR), % | 1.003 (0.993-1.014) | 0.544 |  |  |
| FEV1/FVC, median (IQR), % | 1.017 (0.999-1.035) | 0.070 |  |  |
| Body Mass Index, median (IQR), kg/m^2^ | 0.962 (0.915-1.011) | 0.123 |  |  |
| Red blood cell count, median (IQR), * 10^12/L | 1.304 (1.062-1.602) | 0.011 | 1.401 (1.094-1.794) | 0.008 |
| White blood cell count, median (IQR), * 10^9/L | 1.046 (0.973-1.124) | 0.226 |  |  |
| Hemoglobin, median (IQR), g/L | 1.007 (1.000-1.014) | 0.047 |  |  |
| Platelet, median (IQR), * 10^9/L | 0.999 (0.996-1.001) | 0.353 |  |  |
| Aspartate Transaminase, median (IQR), U/L | 1.028 (1.010-1.047) | 0.002 |  |  |
| Alanine aminotransferase, median (IQR), U/L | 1.013 (0.999-1.027) | 0.075 |  |  |
| Total bilirubin, median (IQR), μmol/L | 1.025 (1.006-1.045) | 0.009 |  |  |
| Direct Bilirubin, median (IQR), μmol/L | 1.036 (1.001-1.073) | 0.046 |  |  |
| Total protein, median (IQR), g/L | 0.994 (0.982-1.006) | 0.345 |  |  |
| Albumin, median (IQR), g/L | 1.009 (0.978-1.041) | 0.591 |  |  |
| Creatinine, median (IQR), μmol/L | 1.002 (0.995-1.009) | 0.583 |  |  |
| Blood urea nitrogen, median (IQR), mmol/L | 1.028 (0.956-1.104) | 0.458 |  |  |
| D-Dimer, median (IQR), mg/L | 1.000 (1.000-1.000) | 0.350 |  |  |
| Total cholesterol, median (IQR), mmol/L | 0.952 (0.817-1.109) | 0.582 |  |  |
| Triglyceride, median (IQR), mmol/L | 0.873 (0.711-1.072) | 0.196 |  |  |
| HDL-C, median (IQR), mmol/L | 0.835 (0.530-1.316) | 0.437 |  |  |
| LDL-C, median (IQR), mmol/L | 0.956 (0.761-1.202) | 0.702 |  |  |
| Erythrocyte sedimentation rate, median (IQR), mm/h | 1.005 (0.998-1.012) | 0.162 |  |  |
| C-reactive protein, median (IQR), mg/L | 1.006 (0.996-1.012) | 0.249 |  |  |
| Partial pressure of carbon dioxide, median (IQR), mmHg | 0.994 (0.985-1.003) | 0.165 |  |  |
| Partial pressure of arterial oxygen e, median (IQR), mmHg | 0.996 (0.985-1.003) | 0.437 |  |  |
| Tricuspid regurgitation velocity, median (IQR), m/s | 2.729 (1.938-3.842) | $<$0.001 | 2.369 (1.645-3.410) | $<$0.001 |
| Echocardiographic PASP, median (IQR), mm Hg | 1.039 (1.002-1.067) | $<$0.001 |  |  |
| Right ventricular diameter, median (IQR), mm | 1.058 (1.032-1.086) | $<$0.001 | 1.036 (1.005-1.068) | 0.021 |
| Right atrial diameter, median (IQR), mm | 1.018 (0.997-1.040) | 0.087 |  |  |
| Left atrial diameter, median (IQR), mm | 0.978 (0.951-1.004) | 0.101 |  |  |
| Pulmonary artery diameter, median (IQR), mm | 1.034 (1.002-1.067) | 0.036 |  |  |
| Ejection Fractions, median (IQR), % | 1.012 (0.990-1.035) | 0.281 |  |  |

Two-tailed *P* values < 0.05 were considered statistically significant.

Abbreviations: CI, confidence interval; FEV1, forced expiratory volume in 1 s; FEV1/FVC%, forced expiratory volume in 1 s/forced volume vital capacity ratio; GOLD, global initiative for chronic obstructive lung disease; HDL-C, high-density lipoprotein cholesterol; IQR, interquartile range; LDL-C, low-density lipoprotein cholesterol; NT-proBNP, N-terminal pro-brain natriuretic peptide; NYHA, New York Heart Association; PASP, pulmonary artery systolic pressure; PH, pulmonary hypertension.

**Table E3. Selected factors in the follow-up cohort for building the prognostic model by univariate and multivariate Cox regression analysis**

| **Variable** | | **Univariate analysis** | | | **Multivariate analysis** | | | | | |
| --- | --- | --- | --- | --- | --- | --- | --- | --- | --- | --- |
|  | | **HR (95% CI)** | ***P* value** | | | | **HR (95% CI)** | ***P* value** | | |
| Sex, No. (%) | |  | 0.878 | | | |  |  | | |
| Male vs. Female | | 0.965 (0.615-1.516) |  | | | |  |  | | |
| Smoking status, No. (%) | |  | 0.992 | | | |  |  | | |
| Ex-smoker vs. Non-smoker | | 1.041 (0.527-2.509) | 0.907 | | | |  |  | | |
| Smoker vs. Non-smoker | | 1.018 (0.639-1.622) | 0.941 | | | |  |  | | |
| Severity of PH, No. (%) | |  | $<$0.001 | | | |  |  | | |
| Severe PH vs. non-severe PH | | 2.553 (1.630-3.999) |  | | | |  |  | | |
| NYHA functional class, No. (%) | |  | 0.008 | | | |  |  | | |
| III vs. I / II | | 1.593 (1.005-2.525) | 0.048 | | | |  |  | | |
| IV vs. I / II | | 2.706 (1.399-5.234) | 0.003 | | | |  |  | | |
| GOLD stage, No. (%) |  | | | 0.101 | |  | | |  |  |
| Stage II vs. Stage I | 2.613 (0.614-11.116) | | | 0.193 | |  | | |  |  |
| Stage III vs. Stage I | 2.619 (0.634-10.821) | | | 0.184 | |  | | |  |  |
| Stage IV vs. Stage I | 3.808 (0.924-15.692) | | | 0.064 | |  | | |  |  |
| Age, median (IQR), year | | 0.992 (0.973-1.012) | 0.419 | | | |  |  | | |
| Smoking exposure, median (IQR), year | | 1.002 (0.991-1.014) | 0.682 | | | |  |  | | |
| NT-proBNP, median (IQR), ng/L | | 1.000 (1.000-1.000) | $<$0.001 | | | | 1.000 (1.000-1.000) | $<$0.001 | | |
| Six-Minute Walk Test, median (IQR), meter | | 0.999 (0.998-1.000) | 0.144 | | | |  |  | | |
| FEV1, median (IQR), % | | 0.990 (0.977-1.003) | 0.117 | | | |  |  | | |
| FEV1/FVC, median (IQR), % | | 1.004 (0.985-1.022) | 0.703 | | | |  |  | | |
| Body Mass Index, median (IQR), kg/m^2^ | | 0.948 (0.898-1.000) | 0.051 | | | |  |  | | |
| Red blood cell count, median (IQR), * 10^12/L | | 1.034 (0.829-1.289) | 0.767 | | | |  |  | | |
| White blood cell count, median (IQR), * 10^9/L | | 1.006 (0.929-1.090) | 0.877 | | | |  |  | | |
| Hemoglobin, median (IQR), g/L | | 1.003 (0.995-1.010) | 0.534 | | | |  |  | | |
| Platelet, median (IQR), * 10^9/L | | 0.995 (0.992-0.998) | 0.004 | | | | 0.995 (0.992-0.999) | 0.005 | | |
| Aspartate Transaminase, median (IQR), U/L | | 1.024 (1.009-1.040) | 0.002 | | | |  |  | | |
| Alanine aminotransferase, median (IQR), U/L | | 1.013 (1.001-1.025) | 0.035 | | | |  |  | | |
| Total bilirubin, median (IQR), μmol/L | | 1.024 (1.009-1.040) | 0.002 | | | |  |  | | |
| Direct Bilirubin, median (IQR), μmol/L | | 1.035 (1.005-1.068) | 0.022 | | | |  |  | | |
| Total protein, median (IQR), g/L | | 1.001 (0.990-1.012) | 0.862 | | | |  |  | | |
| Albumin, median (IQR), g/L | | 0.955 (0.923-0.989) | 0.010 | | | | 0.958 (0.922-0.995) | 0.025 | | |
| Creatinine, median (IQR), μmol/L | | 1.006 (1.001-1.012) | 0.024 | | | |  |  | | |
| Blood urea nitrogen, median (IQR), mmol/L | | 1.047 (0.970-1.129) | 0.237 | | | |  |  | | |
| D-Dimer, median (IQR), mg/L | | 1.000 (1.000-1.000) | 0.142 | | | |  |  | | |
| Total cholesterol, median (IQR), mmol/L | | 1.120 (0.973-1.290) | 0.116 | | | |  |  | | |
| Triglyceride, median (IQR), mmol/L | | 1.042 (0.857-1.268) | 0.680 | | | |  |  | | |
| HDL-C, median (IQR), mmol/L | | 1.079 (0.683-1.705) | 0.743 | | | |  |  | | |
| LDL-C, median (IQR), mmol/L | | 0.864 (0.682-1.095) | 0.228 | | | |  |  | | |
| Erythrocyte sedimentation rate, median (IQR), mm/h | | 0.993 (0.986-1.000) | 0.067 | | | |  |  | | |
| C-reactive protein, median (IQR), mg/L | | 0.996 (0.984-1.007) | 0.454 | | | |  |  | | |
| Partial pressure of carbon dioxide, median (IQR), mmHg | | 1.010 (1.001-1.019) | 0.028 | | | |  |  | | |
| Partial pressure of arterial oxygen, median (IQR), mmHg | | 0.977 (0.964-0.990) | 0.001 | | | | 0.981 (0.968-0.995) | 0.006 | | |
| Tricuspid regurgitation velocity, median (IQR), m/s | | 1.437 (1.051-1.964) | 0.023 | | | |  |  | | |
| Echocardiographic PASP, median (IQR), mm Hg | | 1.011 (1.000-1.021) | 0.047 | | | |  |  | | |
| Right ventricular diameter, median (IQR), mm | | 1.031 (1.006-1.056) | 0.016 | | | |  |  | | |
| Right atrial diameter, median (IQR), mm | | 1.024 (1.004-1.044) | 0.017 | | | |  |  | | |
| Left atrial diameter, median (IQR), mm | | 0.977 (0.947-1.007) | 0.132 | | | |  |  | | |
| Pulmonary artery diameter, median (IQR), mm | | 0.991 (0.961-1.022) | 0.549 | | | |  |  | | |
| Ejection Fractions, median (IQR), % | | 0.999 (0.974-1.025) | 0.956 | | | |  |  | | |
| Mean right atrial pressure, median (IQR), mm Hg | | 1.080 (1.037-1.125) | $<$0.001 | | | |  |  | | |
| Mean right ventricular pressure, median (IQR), mm Hg | | 1.057 (1.036-1.079) | $<$0.001 | | | |  |  | | |
| Mean pulmonary arterial pressure, median (IQR), mm Hg | | 1.051 (1.035-1.067) | $<$0.001 | | | | 1.048 (1.032-1.064) | $<$0.001 | | |
| Pulmonary artery wedge pressure, median (IQR), mm Hg | | 1.035 (0.995-1.078) | 0.090 | | | |  |  | | |
| Cardiac output, median (IQR), L/min | | 0.822 (0.712-0.949) | 0.007 | | | |  |  | | |
| Cardiac index, median (IQR), L/min/m^2^ | | 0.727 (0.575-0.919) | 0.008 | | | |  |  | | |
| Pulmonary vascular resistance, median (IQR), Wood U | | 1.087 (1.046-1.130) | $<$0.001 | | | |  |  | | |
| Total pulmonary resistance, median (IQR), Wood U | | 1.068 (1.029-1.109) | 0.001 | | | |  |  | | |
| Systemic vascular resistance, median (IQR), Wood U | | 0.992 (0.957-1.027) | 0.649 | | | |  |  | | |

Two-tailed *P* values < 0.05 were considered statistically significant.

Abbreviations: CI, confidence interval; FEV1, forced expiratory volume in 1 s; FEV1/FVC%, forced expiratory volume in 1 s/forced volume vital capacity ratio; GOLD, global initiative for chronic obstructive lung disease; HDL-C, high-density lipoprotein cholesterol; HR, hazard ratio; IQR, interquartile range; LDL-C, low-density lipoprotein cholesterol; NT-proBNP, N-terminal pro-brain natriuretic peptide; NYHA, New York Heart Association; PASP, pulmonary artery systolic pressure; PH, pulmonary hypertension.
